# The Spatial Disparities of Land-Use Efficiency in Mainland China from 2000 to 2015

**DOI:** 10.3390/ijerph19169982

**Published:** 2022-08-12

**Authors:** Yunchen Wang, Boyan Li

**Affiliations:** 1 Shaanxi Satellite Application Technology Center for Natural Resources, Shaanxi Institute of Geological Survey, Xi’an 710054, China; 2 Key Laboratory of Spatial-Temporal Big Data Analysis and Application of Natural Resources in Megacities, Ministry of Natural Resources (MNR), Shanghai 200063, China; 3 State Key Laboratory of Information Engineering in Surveying, Mapping and Remote Sensing, Wuhan University, Wuhan 430079, China

**Keywords:** sustainable development goal (SDG) 11.3.1, urban expansion, China

## Abstract

Understanding the sustainable development goal (SDG) 11.3.1-ratio of land consumption rate (LCR) to population growth rate (PGR) is an important prerequisite for planning a guide for sustainable urbanization. However, little is known regarding the degree of accuracy of the estimated LCR due to the inconsistency of data on built-up areas. We extracted four built-up areas, based on inverse S-shaped law and area proportion method, and produced more precise built-up area data (LS_BUA) for the period 2000–2015. Chinese population density data in 2000–2015 was generated based on 26 million points of interest, 19 million roads, other multi-source data, and random forest (RF). Finally, the coupling between LCR and PGR for 340 Chinese cities was calculated during the same period. The results showed that (1) the accuracy of LS_BUA was higher than that of the other built-up area data production methods; (2) the accuracy of test sets in RF exceeded 0.86; (3) the LCR value of mainland China was 0.024 and the PGR value was 0.019 during 2000–2015. The LCR consistently exceeded the PGR and the coordination relationship between LCR and PGR continued to deteriorate. Our research eliminated the difference of SDG 11.3.1 from different data sources and could therefore help decision makers balance land consumption and population growth.

## 1. Introduction

With the acceleration of global urbanization, urban area increased from 362,700 km^2^ to 653,400 km^2^ from 1985–2015 worldwide. The proportion of the global urban population increased from 30% in 1950 to 55% in 2018 [1]. Since the late 2000s, China’s urban built-up area has increased by 78.5%, while the urban population increased by 46% [2]. The complex interaction between human activities (for example, overexploitation of arable land, population urbanization, etc.) and the land system promotes urban expansion and the concentration of human behavior. Farmland, forests, swamps, and green space are the main source of urban expansion. With the acceleration of urbanization, urban expansion poses an increasing threat to cultivated land protection [3]. Therefore, an effective urban planning and governance system must be established [4]. Considering that urban growth is often disproportionate to population growth, it is necessary to accurately identify and continuously monitor long-term urban spatial changes [5].

SDG 11 is defined as Sustainable Cities and Communities, and aims to build inclusive, safe, resilient and sustainable cities with a focus on providing housing security and public transport, sustainable urban development—with an additional focus on preserving world cultures, reducing natural disasters, and improving air quality and provide public spaces, etc. Ratio of land consumption rate (LCR) to population growth rate (PGR) sustainable development goals (SDG 11.3.1) are meant to quantify whether the relationship between the urban built-up area growth and the population growth in a given spatial extent (such as cities, districts, etc.) and time period is coordinated and orderly. The United Nations estimated the LCRPGR of 194 regions and cities around the world and found that the LCR was higher than the PGR in most cities [6]. A global study showed that LCR and PGR showed a downward trend, and the decline in LCR was greater than the decline in PGR in the two periods studied (1975–2000 and 2000–2015) [7]. China’s LCRPGR increased from 1.69 to 1.78 in 1990–2000 and 2000–2010. China has also experienced LCR greater than PGR in many cities. China’s LCR increased, while PGR decreased, from 1990–2010, indicating that the relationship between LCR and PGR was inconsistent. Government control, land policy, household registration system, economic level, and infrastructure are the main reasons for the imbalance and lag between PGR and LCR [8]. However, relevant research only evaluated China’s LCRPGR indicators before 2010, which cannot account for China’s rapid urbanization process [8].

Although the metrics and methods of the SDG 11.3.1 are relatively simple [9], the monitoring of SDG 11.3.1 faces the problem of data inconsistency [10]. Relevant literature and reports have mainly used impervious surface or land cover data to quantify built-up areas (BUA). Timely and accurate monitoring of human settlements (i.e., impervious surfaces area, ISA-land use change data set/source) is essential for understanding the urbanization process [11]. Land cover/land change (LULC) is a complex performance on surfaces covered by natural structures and artificial buildings, and is the natural state of the earth’s surface, such as forests, grasslands, farmland, soil, glaciers, lakes, swamps, wetlands and roads, etc. According to the easy integration of population density data with other spatial data sets and the flexibility in aggregation, the United Nations Human Settlements Program (UN-HABITAT) emphasized that population density data are better than statistics for analyzing spatial heterogeneity [12]. Therefore, generating high-precision urban built-up area and population density data become the essence of accurately estimating SDG 11.3.1.

There are a series of global and regional ISA and LULC data. (1) Impervious surface: 30m global ISA data from 1985 to 2018 were developed by Gong et al. [11], with a set of high-precision long-term impervious surface data. (2) Global urban area data: the commonly global urban area data included three types: Zhou et al. [13], He et al. [14], and Global Human Settlement Layer (GHSL) [15]. The global urban area was alternatively extracted from nighttime light data [16] or was delineated via fully convolutional networks based on the surface temperature, and Normalized Difference Vegetation Index (NDVI) [14]. (3) LULC data: the global LULC data included Globeland 30, Moderate-resolution Imaging Spectroradiometer (MODIS) MCD12 and European Space Agency Climate Change Initiative (ESACCI) data. Globeland 30m was obtained based on the layer-by-layer method in 2000 and 2010 [17]. MCD12 were global LULC data, including three spatial resolutions of 500 m, 1 km, and 0.05 m [18]. The European Space Agency (ESA) released global Climate Change Initiative (ESACCI) data of 300-meter resolution [19]. At the regional scale, China land use data (CLUD) were long time series data, and were extracted by Landsat data and index extraction methods (e.g., Normalized Urban Area Composite Index (NUACI) indicator) [16]. 

All of the above products could be used to extract built-up areas. However, due to the difference between definitions and methods [20], the difference between the estimated global built-up area from different products accounts for 0.45–3% of the total global land area [16]. For example, Gong et al. [11] verified the accuracy of products such as GAIA, GHSL, and Globeland 30 in China and India and found that more built-up areas were identified in products with coarse resolution. Wang et al. [8] treated the urban built-up area statistics obtained by the National Bureau of Statistics of China as true values and compared ESACCI, GHSL, MCD12, and CLUD with statistics in China. They showed that GHSL had the highest relative error, followed by MCD12 data and ESACCI data, while CLUD data had the highest resolution. According to Wang et al., [8]), in 2000, the median relative error of CLUD was less than 1, the median relative error of ESACCI was about 1, the median relative error of MCD12 was about 3, and the median relative error of GHSL was around 6. The built-up area of the above four products (including ESACCI, GHSL, MCD12 and CLUD) were nearly all larger than the statistics of China’s built-up area. Zhang and Zhao [21] found that the MCD12 data were not equivalent to the actual built-up area of Chinese cities, especially in cities with a small built-up area (they retained the core area of the city and eliminated the rural areas in the periphery [22]). In addition, the products of He et al. [14] and Zhou et al. [13] failed to detect significant changes in small built-up areas due to the relatively coarse resolution of nighttime light data. In summary, there were differences between different products in the same research area, especially in cities with smaller built-up areas.

In order to address data inconsistency in the built-up area, the objectives were as follows: we aimed to quantify the spatial differences in land use efficiency in mainland China from 2000 to 2015. Then, the spatial heterogeneity and dynamic trends of LCR, PGR, and LCRPGR in 340 mainland cities were quantitatively analyzed in China from 2000 to 2010 and 2010 to 2015.

## 2. Data Sources

The article used the following data: CLUD data included 6 first-level classifications such as construction land, woodland, etc., with accuracy higher than 75% [16,23]. GAIA data were released by Gong et al. [11], and the average overall accuracy was higher than 90%. We extracted built-up areas based on high-precision GAIA and CLUD, respectively. Defense Meteorological Satellite Program/Operational Linescan System (DMSP/OLS) data and National Polar Orbiting Operational Environmental Satellite System Preparatory Project/Visible Infrared Imaging Radiometer (NPP/VIIRS) data quantitatively recorded global nighttime light intensity. The DMSP/OLS and NPP/VIIRS data were downloaded from National Oceanic and Atmospheric Administration (NOAA). Point of Interest (POI) data were vector data with attributes of position and type (such as tableware, company, etc.). Road data included 7 types of railways, national highways, provincial highways, highways, urban roads, county roads, and village roads. This paper obtained 1,451,271, 4,430,135, and 13,429,160 points of road data, respectively, in 2006, 2010, and 2015 in the study area. We used 2006 POI and road data instead of 2000 due to data availability. MOD13Q1 was a monthly composite of normalized vegetation index (NDVI) data. Elevation data and slope data were obtained through DEM data. The urban built-up area statistics and census come from the Ministry of Housing and Urban-Rural Development of the People’s Republic of China and National Bureau of Statistics, respectively, which were both from the official national authority. Built-up area statistics were used to verify the extracted built-up area accuracy. POIs, DMSP/OLS, NPP/VIIRS and road data provided sources of socio-economic factors for population spatialization, while CLUD, MOD13Q1 and DEM data provided sources of natural factors for population spatialization. Census data, POIs, DMSP/OLS, NPP/VIIRS, road, CLUD, MOD13Q1 and DEM data were used to implement population density mapping. Table 1 details the data sources.

## 3. Methods

### 3.1. The Calculation Process of SDG 11.3.1

SDG 11.3.1 monitors the relationship between the growth rate of the built-up area and the population within a given time and space. The formula [24] is as follows:

LCR = Ln(Urb_t+n_/Urb_t_)/y (1)

PGR = Ln(Pop_t+n_/Pop_t_)/y (2)

LCRPGR = LCR/PGR(3)

where Urb_t+n_ and Urb_t_ represent the built-up area of the current year and the past, respectively. Pop_t+n_ and Pop_t_ represent the current and past population within the built-up area (urban population) respectively. Y represents the interval year. 

**Figure 1:**
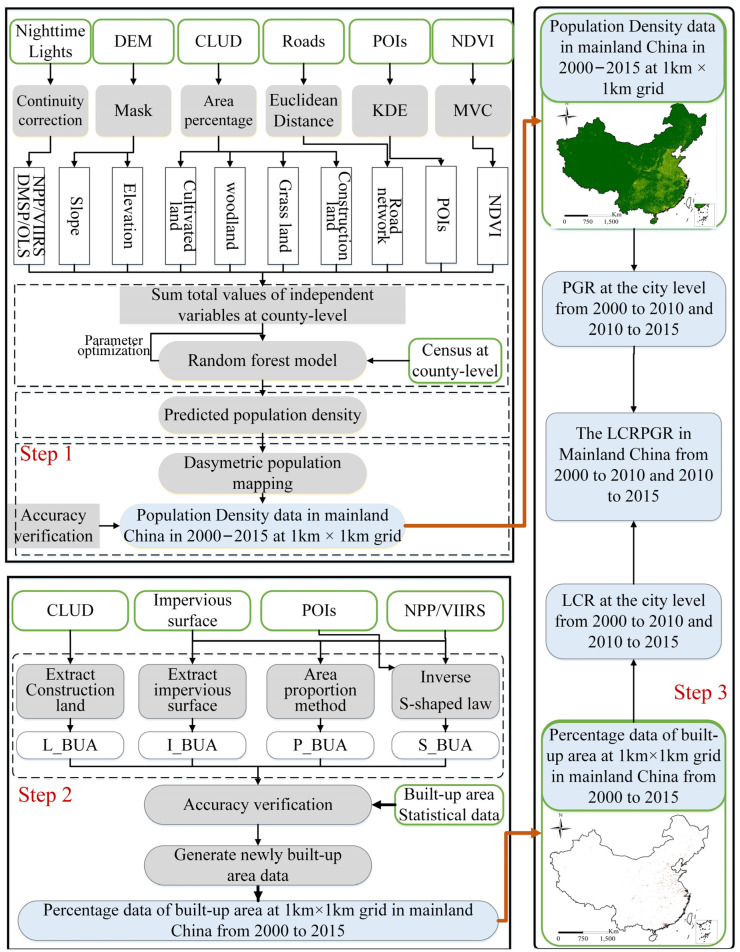
Flowchart. S_BUA and P_BUA data represent a built-up area obtained based on the inverted S-shaped law and area proportion method, respectively. The I_BUA and L_BUA data represent a built-up area obtained from global artificial impervious area (GAIA) and China land cover data (CLUD data), respectively. KDE, kernel density estimation; MVC, maximum value composite.

The SDG 11.3.1 calculation process includes the following three steps (Figure 1):

- Step 1: extracting urban built-up areas. We identified the percentage of urban built-up areas at the level of 1 × 1 km for year 2000, 2010, and 2015 through Section 3.2. Then, the urban built-up area was obtained by aggregating the percentage of the built-up area within the urban administrative boundary.
- Step 2: calculating urban population density data. Through Section 3.3, this paper calculated the population density data of 1 × 1 km in 2000, 2010, and 2015.
- Step 3: calculating SDG 11.3.1. The LCR, PGR, and LCRPGR indicators were calculated according to the data obtained in Step 1 and Step 2 and Equations (1)–(3).

The LCRPGR value was divided into 6 classifications provided by UN-HABITAT (Table S1 in the Supplementary Materials). Considering that the analysis results for different types of cities have greater guiding significance for their development [1], the 340 cities were divided into 5 sizes, according to Table S2 in the Supplementary Materials [8].

### 3.2. The Method of Identified Built-Up Area 

This paper used two methods to extract the built-up area: (1) using GAIA as the data source, we used the inverse S-shaped law (detailed in Section 3.2.1) proposed by [25] to obtain built-up areas (hereafter referred to as ‘S_BUA’ data); (2) ‘P_BUA’ built-up area data were extracted through the area proportion method (detailed in Section 3.2.2) proposed by UN-HABITAT and GAIA data; (3) ‘I_BUA’ built-up areas were determined by directly treating GAIA as a built-up area; (4) we used urban construction land in CLUD as a built-up area (L_BUA data). Then, the new high-precision built-up area percentage was obtained by fusing the built-up area data (LS_BUA).

#### 3.2.1. Identifying Built-Up Areas Based on the Inverse S-Shaped Law

Jiao [25] took the city center point as the circle center and made an equidistant buffer zone from the city center point outward with an interval of 1 km. There was an inverse S law between the ratio of impervious surfaces area (ISA) and the available land area in each circle and the radius. The city was divided into a built-up area, inner-city area, suburban area, and edge according to the law. Domestic cities of different sizes, such as Beijing, Qingdao, Yinchuan, have been used to verify the law. Among them, the goodness of fit (R^2^) was greater than 0.88, and 95% of the cities have R^2^ greater than 0.95, suggesting that the method is suitable for extracting urban built-up areas in large-scale research areas. The formula is as follows:

(4) $$f(r)=\frac{1-c}{1+e^{\alpha (\frac{2r}{D}-1)}}+c$$

where f is the proportion of ISA in the circle, r is the distance to the city center, e is Euler number, α is the slope of the equation curve, c is the ISA density near the city boundary, and D is the radius of the urban built-up area.

#### 3.2.2. Identifying Built-Up Areas through the Area Proportion Method

The method was proposed by UN-HABITAT for extracting the built-up area [26], and requires data sources which can quantify the urbanization degree, with a resolution of 30 m × 30 m or higher resolution. ISA is the main symbol of human settlements, and can quantify the development of urbanization [11]. Therefore, the method used GAIA data as the data source and the steps were as follows:

(1) Calculate the percentage of ISA within a circle of 1 km^2^ around each pixel.
(2) Divide 340 cities into two categories according to the urban population. In the first category, the total urban population must be greater than 50,000; all other cities are placed in the second category [26]. For the first category, the percentage of ISA in pixels is greater than 50 in cities, less than 25% is in rural areas, and the remaining pixels are suburban. For the second category, pixels with ISA percentage greater than 25% are considered cities; otherwise, the pixels are classified as rural.

### 3.3. Mapping the Population Density with RF Model

In this paper, we constructed the nonlinear relationship between the modeling factors and censuses at the county level and transferred the relationship to grid level to achieve census downscaling [27]. Considering natural resources and socioeconomic factors, a total of 10 layers of NDVI, slope, elevation, the area percentage of cultivated, woodland, grassland, construction land, nighttime light intensity data, POI density layer, and road layers, were exploited as independent variables of the model. The independent variables and the census (i.e., dependent variables) were sequentially aggregated at the county level to construct the nonlinear relationship through the RF model. Then, this relationship was transferred to the grid level to obtain population density data. For detailed process, model accuracy and validation of model effects, readers can refer to Wang et al. [27].

### 3.4. Accuracy Assessment

The accuracy of the data was verified by the relative error (RE), root mean square error (RMSE), mean absolute error (MAE), and goodness of fit (R^2^). If R² is closer to 1, the fitting result is better and vice versa. RE, RMSE and MAE can measure the deviation between the observed value and the true value. The smaller the value is, the smaller the deviation. The equation is as follows:

(5) $$RE=\frac{P_{i}-C_{i}}{C_{i}}$$

(6) $$RMSE=\sqrt{\frac{1}{n}{\sum \left(P_{i}-C_{i}\right)}^{2}}$$

(7) $$MAE=\frac{1}{n}\left|P_{i}-C_{i}\right|$$

(8) $$R^{2}=\frac{\sum {\left(P_{i}-\frac{1}{n}\sum C_{i}\right)}^{2}}{\sum {\left(P_{i}-\frac{1}{n}\sum C_{i}\right)}^{2}+\sum {\left(P_{i}-C_{i}\right)}^{2}}$$

where P_i_ and C_i_ are the observed value and the true value, respectively, and n is the number of samples.

## 4. Results

### 4.1. Accuracy Assessment of the Built-Up Area Data

**Figure 2:**
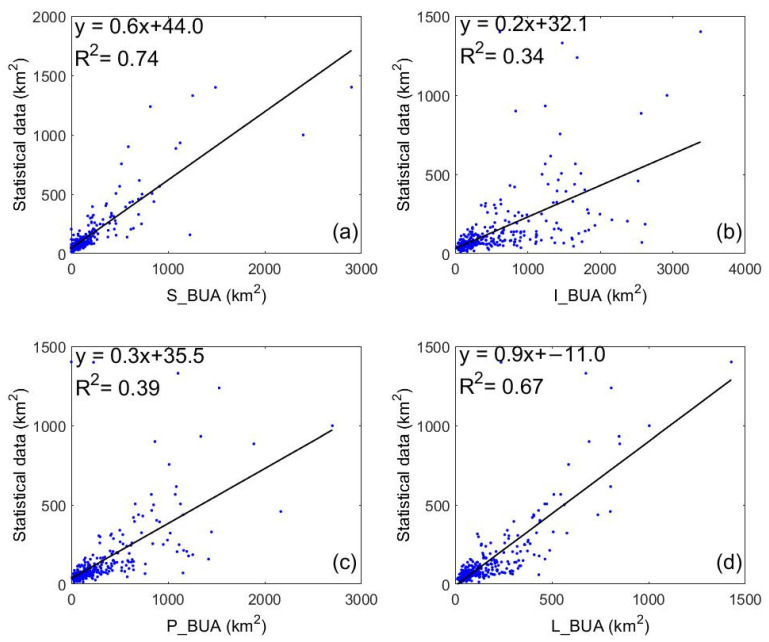
The scatter plot in mainland cities in 2015.

Our research used the built-up area statistics as the dependent variable and the four urban built-up area data (i.e., S_BUA data, P_BUA data, L_BUA data and I_BUA data) as independent variables to calculate the correlation coefficient and R^2^, respectively (Figure 2). Taking 2015 as an example, the correlation coefficient between the statistics and S_BUA was 0.86, which was higher than the other three data, especially in small cities. The correlation coefficient between the statistics and L_BUA was 0.81, R^2^ was 0.67, and the accuracy was lower than S_BUA. The correlation coefficient between statistics and P_BUA was 0.62, R^2^ was 0.39, and the accuracy was significantly lower than the S_BUA and P_BUA. The correlation coefficient between statistics and I_BUA was 0.58, and R^2^ was 0.34, with a clear overestimation. Compared with the built-up area statistics, overestimation often occurs in S_BUA data when the built-up area of a city is greater than 800 km^2^. The P_BUA data results were slightly larger than the statistics. Meanwhile, The I_BUA data results were much larger than the L_BUA data, the S_BUA data, P_BUA data, and the statistics. Therefore, P_BUA and I_BUA will not be analyzed in the future.

**Figure 3:**
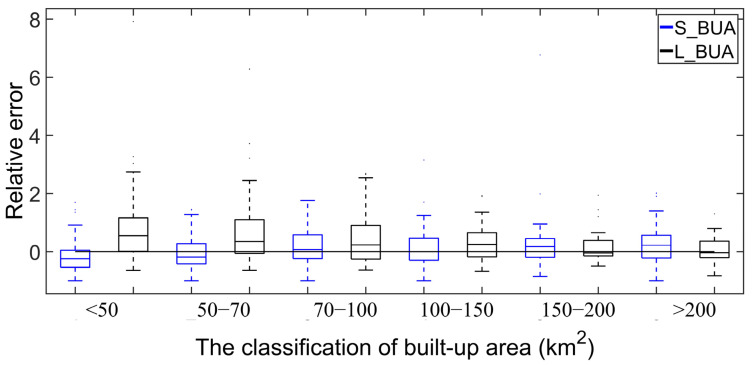
The relative error in 2015.

**Figure 4:**
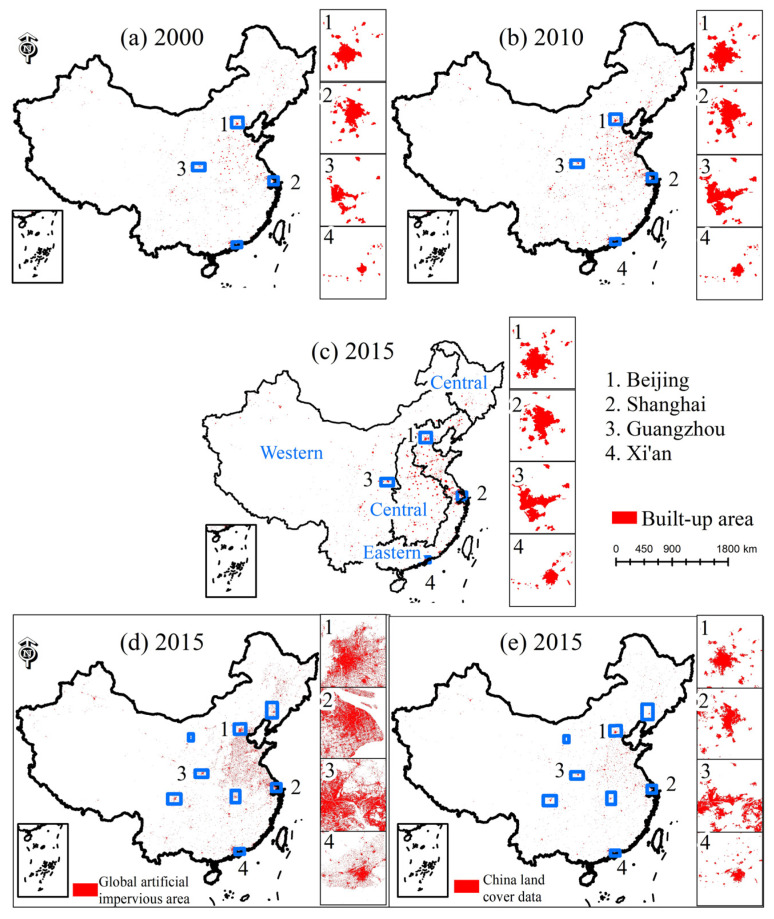
The LS_BUA data, global artificial impervious area (GAIA), and China land cover data (CLUD) in China.

Then, this paper analyzed the RE value of S_BUA and L_BUA data and divided all the cities into 6 categories (Figure 3). Taking 2015 as an example, the RE values of S_BUA and L_BUA were mostly distributed in the −1–1 range, and both had good results. The distribution of RE values of S_BUA data was more concentrated, while the RE value of L_BUA had more extreme values. Specifically, when the built-up area of a city was less than 150 km^2^, the L_BUA data were often overestimated—while the S_BUA data were slightly underestimated. The S_BUA data were closer to 0 and had fewer discrete points than the L_BUA data. When the built-up area of the city was greater than 150 km^2^, the median RE value of the L_BUA data was closer to 0, with fewer discrete points. Therefore, this paper combined cities with built-up areas less than 150 km^2^ in the S_BUA data and cities with built-up areas greater than 150 km^2^ in the current year to obtain the LS_BUA built-up areas in mainland cities from 2000 to 2015 (Figure 4). It can be seen from (c) and (d) in Figure 4 that the urban built-up areas extracted by GAIA and CLUD ere quite different, and the extraction result of the former was significantly larger than that of the latter.

### 4.2. Spatiotemporal Dynamics of LCR

**Figure 5:**
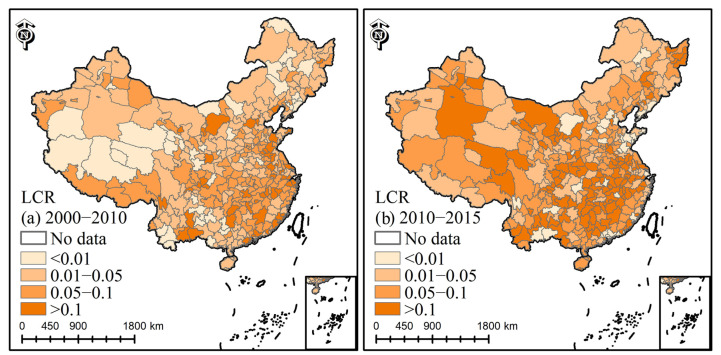
The spatial distribution of land consumption rate (each polygon shape (city) is attached with a color, which represents the city’s LCR value).

The LCR of more than 95% of cities was greater than zero (Figure 5). During 2000–2010, the average LCR of large megacities was 0.055, significantly higher than 2010–2015 (Table 2), and the average LCR of megacities was 0.068, higher than that of the other four city sizes during the same period. Large cities had an average LCR of 0.089 in 2010–2015, which was higher than that of other sizes of cities. The average LCR of small and medium-sized cities was higher than that of large megacities and megacities in 2010–2015. Megacities expanded rapidly during 2000–2015. During this, the expansion of large cities reached its highest rate.

The average values of LCR were: central (0.012) > eastern (0.011) > western (0.010), and the newly increased built-up areas were: 3255.8 km^2^, 10,098.2 km^2^, and 1885.0 km^2^, respectively. From 2010 to 2015, the average LCR values were: central (0.107) > western (0.103) > eastern (0.057), and the growth areas were: 3732.5 km^2^, 2596.7 km^2^, and 4360.9 km^2^, respectively. 

### 4.3. Spatiotemporal Dynamics of PGR

**Figure 6:**
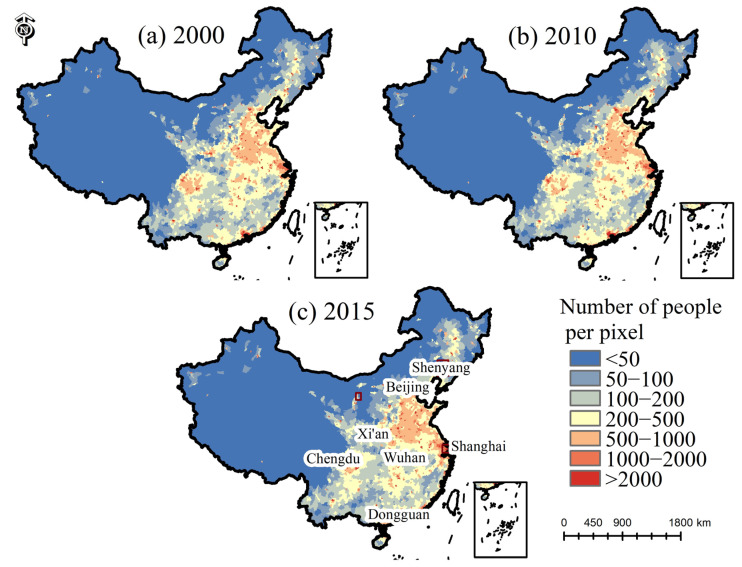
The population density map.

Figure 6 shows the population density classification results. The population distribution in mainland China has an obvious spatial imbalance and further agglomeration since 2000. 

The average PGR of large megacities during 2000–2010 was 0.045, slightly higher than the 2010–2015 period (Table 3). The average PGR of megacities was 0.053 from 2000 to 2010, slightly lower than 2010–2015. Large cities had an average PGR of 0.066 in 2010–2015, which as higher than other sizes of cities in the same period. The average PGR of small and medium-sized cities was the same as that of large megacities during 2000–2015. Notably, during 2010–2015, the population of megacities and large cities grew rapidly.

**Figure 7:**
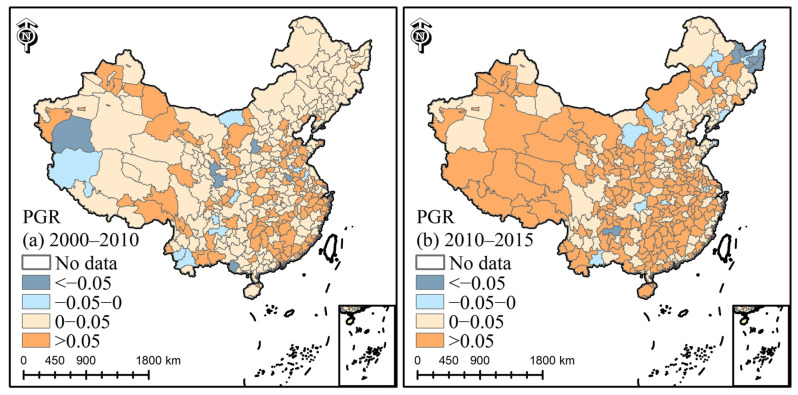
The spatial distribution of population growth rate (each polygon shape (city) is attached with a color, which represents the city’s PGR value).

As shown in Figure 7, the PGR value of Guangdong was higher than that of other cities in 2000–2010. Most cities had PGR greater than 0 with a slow growth rate in 2000–2010. The PGR values of Fujian, Xinjiang, and Inner Mongolia increased in 2010–2015. Although the PGR values of cities such as Beijing were slightly lower than those of western provinces, the actual growth numbers of the urban populations were larger than those of the western population and continue to increase.

In addition, a total of 14 small and medium-sized cities, including Yibin, Dazhou, and Luliang, etc., experienced a decline in urban population in 2000–2010. From 2010 to 2015, 18 small and medium-sized cities, including Hegang, Yichun, Qitaihe, Shuangyashan, and Ordos, also experienced a decline in urban population, and the number of cities increased slightly from the previous period. Among them, the urban population of Bijie continued to decrease from 2000 to 2015. The phenomenon of population decline mostly occurred in small and medium-sized cities. The population outflow from small and medium-sized cities to other cities is not conducive to urban sustainable development.

### 4.4. Spatiotemporal Dynamics of LCRPGR

During the 2000–2015 period, the average LCR and PGR values of large megacities decreased at the same time, while the LCR value decreased faster (Table 4). For example, Guangzhou and Shenzhen were the two first-tier cities in the Pearl River Delta, one of the most economically developed regions in China. In the two periods of 2000–2010 and 2010–2015, Guangzhou’s LCR increased, but Shenzhen’s LCR decreased. The average LCR of the megacities decreased, while the PGR increased slightly in the two periods. In 2000–2015, both LCR and PGR of large cities increased. In contrast, the LCR value of small and medium-sized cities experienced growth from 2000 to 2015 (e.g., Lasa) because the expansion of western cities accelerated slightly—and due to heavy losses in population.

**Figure 8:**
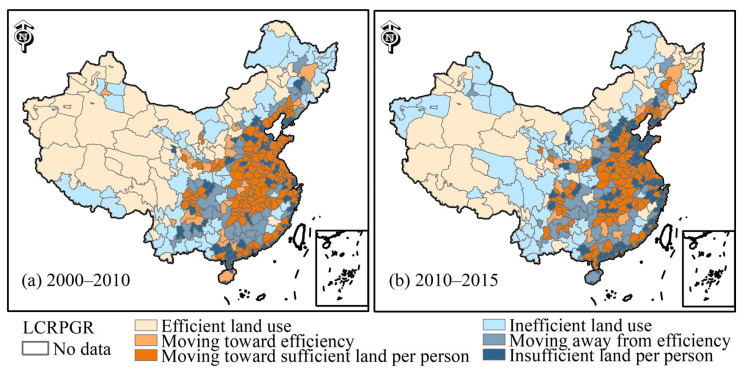
The spatial distribution of the ratio of land consumption to population growth rate (each polygon shape (city) is attached with a color, which represents the city’s LCRPGR type).

Figure 8 shows that 128 (37.6%) cities were moving toward sufficient land per person type during 2000–2010. The data were mainly distributed in cities along the southeast coast. Only 24 (7.1%) cities were moving toward efficiency, mainly in Gansu and Shaanxi. Additionally, 51 (15%) cities belonged to the effective land use type and were distributed in Xinjiang and Tibet. A total of 43 cities fell into insufficient land per person type with high population densities. For example, the PGR was significantly higher than LCR in first-tier cities such as Beijing. Furthermore, 40 (11.7%) cities are moving away from efficiency, such as Chongqing. Additionally, 54 (15.8%) cities had inefficient land use, such as Yichun. Its LCR showed positive growth, and the PGR showed negative growth, leading to an inconsistent relationship between the LCR and PGR.

From 2010 to 2015, there were 107 (31.4%) cities in the category of moving toward sufficient land per person, a decrease from the previous period. Some cities actually changed to show insufficient land per person. For example, the LCR and PGR values of Chengdu both declined, and the LCR value decreased more rapidly. A total of 19 (5.5%) cities were moving toward the efficiency type, less than in the previous period. There were 45 (13.2%) cities with efficient land use types. For example, the LCR and PGR increased in the Qamdo area of Tibet during both periods, but the LCR was faster than the PGR. There ere 67 (19.7%) cities with insufficient land per person, mainly distributed in Qingdao, Dongying, and other cities with higher population density. A total of 45 (13.2%) cities were moving away from efficiency in land use, such as Ganzhou. There were 57 (16.7%) inefficient land-use cities distributed in low population density areas, such as Xinjiang and Tibet in the west. In summary, the number of cities with efficient land use, moving toward efficiency, and moving toward sufficient land per person type decreased from 203 (59%) to 171 (51%), and the number of cities with inefficient land use, moving away from efficiency, and insufficient land per person type cities increased from 137 (41%) to 169 (49%) between 2000–2010 and 2010–2015. The LCR value was 0.024, the PGR value was 0.019, and the LCRPGR value was 1.27 in 2000–2015.

## 5. Discussion

### 5.1. Verification Based on Existing Research

This article made a comparative analysis with previous research results due to lack of truth-value. We found that China’s newly built-up area was 25,929 km^2^, and the urban population increased by 110 million from 2000 to 2015. China’s built-up area increased by 2.6 times, and the urban population increased by 1.9 times. Wang et al. [8] indicated that, from 1999 to 2010, China’s built-up area increased 2.5-fold, and the urban population increased 1.7-fold. Schneider and Mertes [28] showed that, from 1978 to 2010, China’s built-up area tripled, and the population doubled. Our research results were consistent with previous results.

Our study revealed that the LCR value was 0.024, the PGR value was 0.019, and the LCRPGR value was 1.27 from 2000–2015, values which were found to be similar to those in other contexts [8]. During the 1990–2015 period, the LCR values of 10,000 cities worldwide were 1.2 times higher than the PGR during 1990–2015 [29]. The LCRPGR value of sample cities in developed countries was 1.9 from 2000 to 2015 [6]. Meanwhile, the United Nations [6] estimated the LCRPGR of 194 regions and cities around the world and found that the LCRPGR was 1.74 from 2000 to 2015. The LCRPGR value of this paper was lower than that of developed countries because China’s urbanization is in a period of formation at the time of this writing [8]. In addition, during the two periods (1975 to 2000 and 2000 to 2015), the report showed that the global LCR and PGR trended downward, but the decline of LCR was greater than that of PGR [7]. This was also reflected in China’s megacities.

### 5.2. The Accuracy of Built-Up Area and Population Density Data

#### 5.2.1. The Accuracy of Built-Up Area 

**Figure 9:**
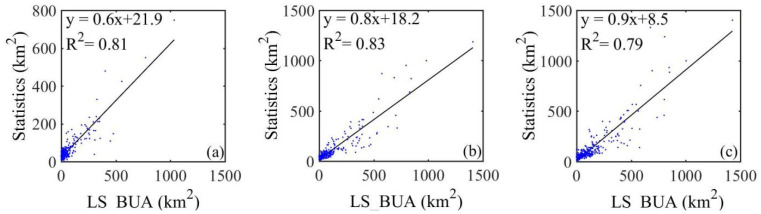
The scatter plot of LS_BUA and built-up area statistics in (**a**) 2000, (**b**) 2010, and (**c**) 2015.

To verify the accuracy of built-up area data, this paper utilized the statistics as the dependent variable and the S_BUA, P_BUA, I_BUA, L_BUA, and LS_BUA data as independent variables to calculate the correlation coefficients and R^2^. We also calculated the MAE and RMSE between the statistics and the aforementioned data. From 2000 to 2015, the correlation coefficients between built-up area statistics and LS_BUA data were 0.9, 0.91, and 0.88, while the R^2^ values were 0.81, 0.83, and 0.79, respectively (Figure 9). As shown in Table 5 and Table 6, from 2000 to 2015, the RMSE values of LS_BUA data were 80.9, 74.9, and 89.0, and the MAE values were 47.2, 45.1, and 51.8, respectively. These were all smaller than the other four built-up areas. Therefore, the LS-BUA data were higher than the other four types.

The expansion of urban ISA is an important part of rapid urbanization. However, the spectral characteristics of vegetation, soil, and ISA are partially similar, leading to overestimation of small areas and underestimations of large areas [11]. Due to conflicts of interest in land use planning, small-scale changes in city centers and changes at the edges of a city will also result in overestimation or underestimation in the quantification of ISA [30]. ISA and LUCC data are larger than the statistical value, and it is possible that: (1) the presence of a large number of mixed pixels in the image could lead to misclassification during decomposition [28]; (2) high built-up areas could derive from misclassified non-vegetation land in the suburbs and squares, parks, green spaces, and roads in city core area due to resolution limitation. Moreover, the built-up area data required for the land consumption rate in the SDG 11.3.1 metadata differ from the concept of ISA or construction land data in land use data [8]. Through a survey of 231 cities around the world, United Nations found that 59% of urban built-up areas contained public space [12]. The heterogeneity was mainly due to the definition of public space, including parks, gardens, and roads, which affected the estimation of built-up areas. In fact, the extraction of built-up areas relies more on data sources or training samples rather than semantic definitions, resulting in differences in depicting the boundaries of built-up areas [31]. 

#### 5.2.2. The Accuracy of Population Density Data

This article used the RF method to build a population density model. This paper randomly selected 70% of the input data set as a training set for training model. The remaining 30% was the test set to calculate the model accuracy. The training set accuracy exceeded 0.97 (Table 7). The variability percentage of the dependent variable, which can be explained by the RF regression model, was above 0.86.

**Figure 10:**
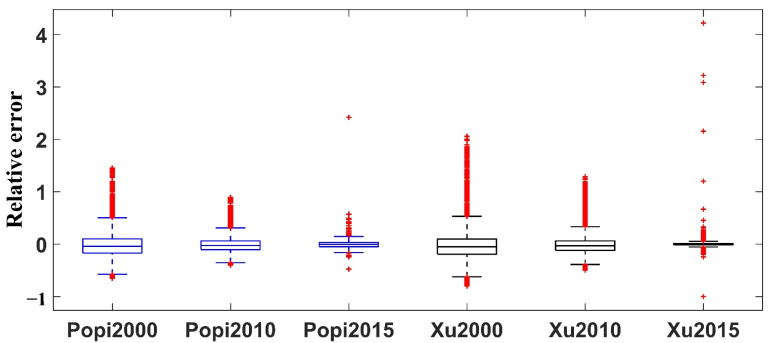
The relative error of population density data and census.

To verify the accuracy of the population density data, this paper separately summarized the total population values in the administrative units of Popi and Xu [32] population density data, respectively. Xu [32] considered the land use, nighttime light data, density of residential areas, and other factors, then used the multifactor weight distribution method to spread the population data on the 1 × 1 km level in mainland China from 2000 to 2015. The results were released at https://www.resdc.cn/DOI/DOI.aspx?DOIid=32 (accessed on 2 March 2021). We found that both Popi data and Xu [32] had good accuracy in 2000–2015 (Figure 10). Compared with Xu [32], the median of the RE value of Popi data was close to 0, with fewer discrete points. We found that the proportion of cities with an absolute RE < 0.25 of the two data exceeded 0.96, and the proportion of cities with an absolute RE > 0.5 of the Popi data was slightly smaller than that of Xu [32] (Table 8). Popi was more advantageous because the Popi data integrated more than 20 million geographic Big Data points from POIs and roads, which were closely related to the population. Additionally, the RF nonlinear model was more suitable for estimating population density [27].

### 5.3. The Analysis of LCR

There were differences in LCR in different regions. The eastern region was in an advantageous position in terms of location, economy, and policies. In 1984, 14 cities in the east were designated as coastal cities. Since then, the Yangtze River Delta, the Pearl River Delta, and the Xia-Zhang-Quan Delta have been regarded as coastal economic open areas. Coastal open cities have good conditions, e.g., geographic location, natural resources, economic foundation, and technical management. For example, in 2000–2010, the LCR value of Dongguan reached 0.23. In general, the land use efficiency values in eastern cities were higher, promoting urban intensification and slowing down urban expansion. In the central region, the LCR values of cities such as Zhengzhou, Changsha, Hefei, and Luoyang gradually increased. The central region had a larger area and more available land. Therefore, the LCR values of the central region were slightly larger than those of the eastern region. Except for Chengdu, Chongqing, Lhasa, Kunming, and Xi’an, most cities in the western region were slowly expanding. Since the Western Development Policy in 2000, the process of industrialization in many cities has accelerated. As capital cities, Chengdu and Xi’an have played a radiating and leading role in southwest and northwest China. Overall, urban expansion concentrated in the eastern coastal region, while the growth in the central and western regions was mainly reflected in capitals.

Due to differences in factors such as social economy, regional location, and policy orientation, there were differences in LCR indicators between the southeast coastal cities. For example, Guangzhou and Shenzhen were the two first-tier cities in the Pearl River Delta located in the southeast coastal region, one of the most economically developed regions in China. In the two periods of 2000–2010 and 2010–2015, Guangzhou’s LCR increased, and Shenzhen’s LCR decreased. Likely explanations for these dynamics could be as follows: firstly, it has. been restricted with administrative division. The administrative division area in Guangzhou was approximately 3.7 times than that of Shenzhen, and the available land resources were relatively abundant. Secondly, it was restricted in terms of the topography and geographical location. The hilly area of Shenzhen was larger than that of Guangzhou. The terrain of Guangzhou and surrounding was flat, and thus conducive to the formation of an economic belt in Guangfo City with Foshan in the west. Thirdly, it was restricted with supply chain industries. Shenzhen’s unique position and preferential policies have given it competitive advantages in innovation and technology and accelerated the city’s supply chain access to the Chinese mainland market. However, infrastructure provided by Guangzhou was more abundant than that of Shenzhen, resulting in more urban expansion in Guangzhou. In addition, Dongguan’s was significantly faster than other cities in the Pearl River Delta, such as Zhaoqing, Huizhou, and Jiangmen. Likely explanations for these differences could be as follows: first, Dongguan was one of the fastest-expanding cities in China during the periods of study. Industrial development is one of the most important factors driving development of China’s land. Rapid industrial development requires a large amount of land, which drove the observed rapid expansion of Dongguan. Second, driven by the radiation of first-tier cities, adjacent cities expanded rapidly. The proportion of forest land in Zhaoqing (69%), Huizhou (60%), and Jiangmen (46%) were relatively high, going against the expansion of built-up areas. The above three cities were also far from economically developed cities such as Guangzhou, and thus, their urban expansion rates were slow. 

### 5.4. Ratio of Land Consumption Rate to Population Growth Rate

The LCR values of many cities in China were higher than the PGR in the same period due to rapid urban expansion and an aggravated imbalance between built-up areas and population. For example, the built-up area of Dongguan city on the southeast coast more than tripled from 2000–2015. There are several possible reasons for this. First, in terms of external systems. The dual land system (urban land is owned by the state, and rural land is owned by the collective) proposed in *The Constitution of the People’s Republic of China* and *the Land Administration Law of the People’s Republic of China* led to the formation of two markets for agricultural land and nonagricultural land in China’s land market (https://baike.baidu.com/item/%E5%9F%8E%E4%B9%A1%E4%BA%8C%E5%85%83%E4%BD%93%E5%88%B6%E6%94%B9%E9%9D%A9/7970059 (accessed on 2 March 2021)). Low land acquisition costs and rapid industrial development transformed a large number of agricultural land markets into nonagricultural land markets, driving the growth of the LCR [7]. At the same time, the dual urban-rural household registration system (in which household registration is divided into agricultural household and nonagricultural household) proposed in *Regulations of the People’s Republic of China on Household Registration* had a depressive effect on the PGR. People with rural household registration used to live in rural areas. Urban expansion needs to absorb more rural people, but it cannot provide them with necessary living conditions, such as employment, medical care, education, and housing, slowing down urban population growth. 

Second, we considered internal mechanisms. To attract investment, the price of industrial land is usually lowered, leading to an increase in urban LCR. However, the price of residential land has increased, increasing the cost of population migration and inhibiting the PGR. This leads to a phenomenon in which farmers’ land has been urbanized, but farmers have difficulty integrating into the city. 

Third, we considered urban planning. Attracting investment and generating income based on land sales is a common policy tool in China [33]. As the speed of urban land construction and planning is not coordinated, there are phenomena such as vacant, wasted urban land and unreasonable spatial distribution. For example, the phenomenon of land fences, excess land supply, and undeveloped land that will further accelerate the expansion of built-up areas and the inefficiency of urban land use [4]. In summary, factors such as government control, land policy, household registration system, economic level, and infrastructure are the main reasons for the imbalance and lag between PGR and LCR.

Other factors that affect LCRPCR included national or local development plans, priorities and policies, and local socioeconomic issues, which will require more concentrated research and analysis. For example, the high PGR in developed cities in the Pearl River Delta resulted mostly from the population flow after the reform and opening policy since 1978. Population growth, per capita income, and informal settlements were the main driving forces behind rapid urban expansion [6]. The increase in migrant workers in the Pearl River Delta region could lead to an increase in local PGR, and the relationship between LCRPCR may grow more complicated. In addition, we found that some cities had an extreme LCRPGR value greater than 5 or less than −5. It was also determined that 143 (27%) cities in Eurasia had an extreme LCRPGR value from 2000 to 2015 [1]. These extreme values may lead to small but important changes in the PGR indicator over time, based on the definition and formula of the LCRPGR indicator.

Finally, the living space needed to accommodate the population may be obtained through urban space expansion or be solved through urban vertical development, as seen in Hong Kong and Lanzhou City, China. Hong Kong has been limited by administrative division areas, while Lanzhou was developed by an extension of the river valley. The area of land available for development in both cities has been and remains restricted. The LCRPGR indicator cannot quantify the relationship between urban three-dimensional spatial expansion and population growth, as it ignores the third dimension of urban geography, that is, the impact of height information on urban expansion. Frantz et al. [34] and Li et al. [35] exploited Sentinel-1, Sentinel-2 and other auxiliary data to generate building height data, providing data support for the evaluation of SDG 11.3.1 on a three-dimensional level. It could further satisfy the interpretation and analysis of the LCRPGR index on a vertical scale. However, there are still many challenges in generating large-scale building height data.

## 6. Conclusions

Our research used remote sensing image data, statistics, and geographic Big Data to calculate the percentage of built-up area and population density data at 1 × 1 km grid level in mainland China in the period 2000–2015. It also analyzed the temporal and spatial relationships, evolutionary laws, and formation reasons of LCRPGR value in 340 cities from 2000 to 2015. The conclusions were as follows:

(1) The accuracy of the LS_BUA as higher than the other four built-up area data types. The RMSE values of the LS_BUA in 2000, 2010, and 2015 were 80.9, 74.9, and 89.0, respectively, and the MAE values were 47.2, 45.1, and 51.8 respectively;
(2) In 2000–2015, the test set accuracy exceeded 0.86, and the proportion of cities with an absolute value of RE of Popi data < 0.25 exceeded 0.96;
(3) The number of inefficient land use, moving away from efficiency, and insufficient land per person type cities increased from 137 (41%) to 169 (49%) between 2000–2010 and 2010–2015. The PGR value was 0.019, and the LCRPGR value was 1.27. The LCR consistently exceeded the PGR and the coordination relationship between LCR and PGR continued to deteriorate.

Our research could be extended to other countries for accurate estimated SDG indicators, realizing the long-term quantitative analysis of urbanization processes. As such, our research could also be essential for guiding decision-makers to balance land consumption and population growth.

## Figures and Tables

**Table 1:** Data Sources.

Data	Resolution/Format	Time	Sources
China land cover data, (CLUD)	100 m	2000, 2010, 2015	Data Centre for Resources and Environmental Sciences (http://www.resdc.cn/data.aspx?DATAID=99), (accessed on 2 March 2021).
Global artificial impervious area, (GAIA)	30 m	2000, 2010, 2015	Tsinghua University (http://data.ess.tsinghua.edu.cn), (accessed on 2 March 2021).
Defense Meteorological Satellite Program/Operational Linescan System (DMSP/ OLS)	1 km	2000, 2010	National Oceanic and Atmospheric Administration, NOAA (https://www.ngdc.noaa.gov/eog/dmsp/downloadV4composites.html), (accessed on 2 March 2021).
National Polar Orbiting Operational Environmental Satellite System Preparatory Project/Visible Infrared Imaging Radiometer (NPP/ VIIRS)	750 m	2015	NOAA (https://ngdc.noaa.gov/eog/viirs/download_dnb_composites.html), (accessed on 2 March 2021).
Normalized Difference Vegetation Index (NDVI)	250 m	2000, 2010, 2015	Moderate Resolution Imaging Spectroradiometer, MOD13Q1 (http://ladsweb.modaps.eosdis.nasa.gov/), (accessed on 2 March 2021).
Digital Elevation Model (DEM)	90 m	2000	National Aeronautics and Space Administration, NASA (http://srtm.csi.cgiar.org/srtmdata/), (accessed on 2 March 2021).
Point of Interests (POIs)	Point features	2006, 2010, 2015	Baidu Maps API (http://lbsyun.baidu.com/), (accessed on 2 March 2021).
Roads	Line features	2006, 2010, 2015	Baidu Maps API (http://lbsyun.baidu.com/), (accessed on 2 March 2021).
Built-up area	City	2000, 2010, 2015	2001, 2011 and 2016 China Urban Construction Statistical Yearbook (http://www.mohurd.gov.cn/xytj/tjzljsxytjgb/jstjnj/), (accessed on 2 March 2021).
Census	County, City	2000, 2010, 2015	The Fifth and Sixth National Population Census, National Bureau of Statistics of China (http://www.stats.gov.cn/tjsj/pcsj/), 2016 Statistical Yearbook of Provinces and Cities, National Bureau of Statistics of China (http://www.stats.gov.cn/tjsj/pcsj/), (accessed on 2 March 2021).
Administrative boundary map	County, City	2013	National Fundamental Geography Information System (http://www.ngcc.cn/ngcc/), (accessed on 2 March 2021).

**Table 2:** The classification average results of land consumption rate.

City Size	LCR		
	2000–2010	2010–2015	2000–2015
Large Megacities	0.055	0.017	0.041
Megacities	0.068	0.066	0.067
Large cities	0.047	0.089	0.064
Medium cities	0.043	0.077	0.054
Small cities	0.033	0.077	0.046

**Table 3:** The classification results of population growth rate.

City Size	PGR		
	2000–2010	2010–2015	2000–2015
Large Megacities	0.045	0.044	0.045
Megacities	0.053	0.062	0.056
Large cities	0.040	0.066	0.049
Medium cities	0.042	0.041	0.042
Small cities	0.048	0.044	0.045

**Table 4:** The classification results of LCRPGR.

City Size	LCRPGR		
	2000–2010	2010–2015	2000–2015
Large Megacities	1.310	0.292	1.064
Megacities	1.828	0.629	1.508
Large cities	1.453	0.156	1.428
Medium cities	1.212	1.654	1.107
Small cities	0.466	1.756	1.037

**Table 5:** The RMSE values of built-up area data (km^2^).

	S_BUA	P_BUA	I_BUA	L_BUA	LS_BUA
2000	90.7	112.2	393.1	97.0	80.9
2010	135.3	219.3	550.9	126.0	74.9
2015	197.0	390.1	756.2	139.8	89.0

**Table 6:** The MAE values of built-up area data (km^2^).

	S_BUA	P_BUA	I_BUA	L_BUA	LS_BUA
2000	49.1	53.4	215.5	52.7	47.2
2010	50.6	102.9	305.8	63.6	45.1
2015	73.1	198.4	437.3	67.4	51.8

**Table 7:** The goodness of fit (R^2^) values of the RF model.

R^2^	2000	2010	2015
Training set accuracy	0.97	0.98	0.98
Test set accuracy	0.86	0.87	0.88

**Table 8:** The relative error between the population density data and census.

Relative Error	Popi			(Xu, 2017 [32])		
	2000	2010	2015	2000	2010	2015
>0.5	1.21%	0	0.31%	2.10%	0.58%	0.59%
0.25–0.5	0.60%	0	2.79%	0.60%	0.29%	2.67%
−0.25–0.25	96.77%	99.05%	96.56%	96.38%	98.52%	96.42%
−0.5–−0.25	1.51%	0.94%	0.31%	0.60%	0.29%	0
<−0.5	0	0	0	0.30%	0.29%	0.29%

## Data Availability

Land use/land cover (LULC) datasets were gained from the Data Centre for Resources and Environmental Sciences (RESDC) web page http://www.resdc.cn/data.asp x?DATAID = 99 (accessed on 2 March 2021). Global artificial impervious area (GAIA) were downloaded from Tsinghua University (http://data.ess.tsinghua.edu.cn) (accessed on 2 March 2021). DMSP/OLS were gained from National Oceanic and Atmospheric Administration, NOAA (https://www.ngdc.noaa.gov/eog/dmsp/downloadV4composites.html) (accessed on 2 March 2021). NPP/VIIRS were downloaded from NOAA (https://ngdc.noaa.gov/eog/viirs/download_dnb_composites.html) (accessed on 2 March 2021). NDVI were gained from Moderate Resolution Imaging Spectroradiometer, MOD13Q1 (http://ladsweb.modaps.eosdis.nasa.gov/) (accessed on 2 March 2021). DEM were downloaded from National Aeronautics and Space Administration, NASA (http://srtm.csi.cgiar.org/srtmdata/) (accessed on 2 March 2021). POIs were downloaded from Baidu Maps API (http://lbsyun.baidu.com/) (accessed on 2 March 2021). Roads were downloaded from Baidu Maps API (http://lbsyun.baidu.com/) (accessed on 2 March 2021). Built-up area were downloaded from China Urban Construction Statistical Yearbook (http://www.mohurd.gov.cn/xytj/tjzljsxytjgb/jstjnj/) (accessed on 2 March 2021). Census were downloaded from The Fifth and Sixth National Population Census, National Bureau of Statistics of China (http://www.stats.gov.cn/tjsj/pcsj/) (accessed on 2 March 2021), and 2016 Statistical Yearbook of Provinces and Cities, National Bureau of Statistics of China (http://www.stats.gov.cn/tjsj/pcsj/) (accessed on 2 March 2021). Administrative boundary map were downloaded from National Fundamental Geography Information System (http://www.ngcc.cn/ngcc/) (accessed on 2 March 2021).

## Supplementary Materials

The following supporting information can be downloaded at: https://www.mdpi.com/article/10.3390/ijerph19169982/s1, Table S1: The classification criteria; Table S2: The whole list of 340 cities.
