# Supplementary material for: The Spatial Disparities of Land-Use Efficiency in Mainland China from 2000 to 2015
Source: Int J Environ Res Public Health. 2022 Aug 12;19(16):9982. doi: 10.3390/ijerph19169982 (PMC9408634; doi:10.3390/ijerph19169982)
Supplement: Supplementary file 1 [file ijerph-19-09982-s001.zip › ijerph-1838309-supplementary.pdf]

Supplementary Information for

The Spatial Disparities of the Land-Use Efficiency in Mainland China from 2000 to 2015

Summary of Supporting Information

Table S1. The classification criteria.

City Urban Extent Density	LCRPGR Value	Code
10–150 persons/hectare	<1: Efficient land use	1
	>1: Inefficient land use	2
151–250 persons/hectare	<1: Moving toward efficiency	3
	>1: Moving away from efficiency	4
>250 persons/hectare	<1: Insufficient land per person	5
	>1: Moving toward sufficient land per person	6

Table S2. The whole list of 340 cities.

City Sizes	Population	Number of Cities	City
Large megacities	>10 million	13 (3.8%)	Beijing, Shanghai, Tianjin, Guangzhou, Chengdu, Shenzhen
Megacities	5 million–10 million	77 (22.6)	Chongqing, Dongguan, Soochow, Foshan, Shijiazhuan, Nanjing, Wuhan, Shenyang, Wenzhou, Hangzhou, Harbin, Wuxi, Qingdao, Weifang, Quanzhou, Baoding
Large cities	1 million–5 million	214 (62.9%)	Jinan, Tangshan, Ningbo, Dalian, Xi'an, Nantong, Changsha, Yantai, Linyi, Changchun, Zhongshan, Handan, Nanchang, Taiyuan, Fuzhou, Yancheng, Ganzhou, Xuzhou, Langfang, Changzhou, Kunming, Huizhou, Hefei, Cangzhou, Shan Tou, Zibo, Urumqi, Taizhou, Zhengzhou, Hengyang, NanNing, Dezhou, Xingtai, Lan'Zhou, Jiangmen, Binzhou, Jieyang, Datong, Xiamen, Anshan, Shaoxing, Tai'an, Shangrao, Zhangjiakou, Yangzhou, Taizhou, Baotou, Liaocheng, Zhangzhou, Jinhua, Dongying, Mianyang, Jilin, Jining, Jiaxing, Zhuzhou, Zhangjiang, Deyang, Qiqihar, Luoyang, Yueyang, Lianyungang, Nanchong, Shaoyang, Yulin, Hohhot, Zunyi, Guiyang, Qujing, Ji'an, Kashgar Prefecture, Zhenjiang, Jinzhong, Maoming, Huaian, Qinhuangdao, Suqian, Hengshui, Chengde,

			Huanggang, Loudi, Liuzhou, Jinzhou, Weihai, Suihua, Ili Kazakh Autonomous Prefecture Yongzhou, Hulunbeir, Meizhou, Yingkou, Shengzhou, Zhuhai city, Qingyuan, Luliang, Yibin, Hani-Yi Autonomous Prefecture of Honghe, Dali Bai Autonomous Prefecture, Fuzhou, Xiangtan, Zhaoqing, Chifeng, Longyan, Daqing, Anyang, Yichun, Guilin, Jingzhou, Yulin, Xinzhou, Xining, Wenshan Zhuang and Miao Autonomous Prefecture, Yinchuan, Changde, Aksu Prefecture, Zaozhuang, Yiyang, Fushun, Bijie, Meishan, Siping, Ulanqab, Chaozhou, Neijiang, Yangjiang, Heze, Dazhou, Mudanjiang, Yanbian Korean Autonomous Prefecture, Dandong, Changzhi, Haikou, Jiujiang, Huludao, Xinxiang, Huaihua, Ziyang, Jiamusi, Shaoguan, Heyuan, Yichang, Ordos, Huzhou, Shuozhou, Anqing, Tongliao, Shengzhou, Linfen, Liaoyang, Lincang, Yuncheng, Wuhu, Liangshan Yi Autonomous Prefecture, Rizhao, Jingdezhen, Zhaotong, Leshan, Xianyang
Medium cities	500,000–1,000,000	20 (5.8%)	Zigong, Baoji, Putian, Tieling, Baoshan, Yangquan, Huangshi , Chaoyang, Pu'er, Dingxi, Panjin, Bayingol Mongolian Autonomous Prefecture, Tonghua, Xiaogan, Ningde, Jixi, Hechi, Shanwei, Baise, Bazhong, Bayan Nur, Fuyang, Songyuan, Nanping, Fuxin, Wuzhou, Baicheng, Suining, Xuancheng, Guang'an, Tianshui, Shiyan, Suzhou, Benxi, Maanshan, Lu'an, Qiandongnan Miao and Dong Autonomous Prefecture, Shengzhou, Laiwu, Hegang, Yuxi, Chuxiong, Changji Hui Autonomous Prefecture, Guigang, Anshun, Qiannan Buyi and Miao Autonomous Prefecture, Weinan, Baiyin, Jiaozuo, Shengzhou, Heihe, Pingxiang, Yunfu, Sanming, Liyang, Xianning, Tarbagatay Prefecture, Sanya, Liupanshui, Hotan Prefecture, Hanzhong, Wuhai, Xiangyang, Wuwei, Lishui, Tongren, Southwest Guizhou Autonomous Prefecture, Xinyu, Yichun, Shengzhou, Bengbu, Guangyuan, Xilingol League, Enshi Tujia and Miao Autonomous Prefecture, Pingdingshan, Kaifeng,Qinzhou, Qitaihe, Sanmenxia, Shizuishan, Shangqiu, Hinggan League, Baishan, Wuzhong, Huainan, Jincheng, Ya'an, Huaibei, Shuangyashan, Huangshan, Hami, Beihai
Small cities	<500,000	16 (4.7%)	Hezhou, Pingliang, Xuchang, Tongling, Dehong Autonomous Prefecture, Xinyang, Lhasa, Jiuquan, Liaoyuan, Zhoukou, Zhangye, Jingmen, Xiangxi Tujia and Miao Autonomous Prefecture, Linxia Hui Autonomous Prefecture, Lijiang, Guyuan, Zhongwei, Suizhou, Longnan, Chizhou, Qingyang, Jinchang, Yan'an, Panzhihua, Hebi,

	Laibin, Kelamayi, Yingtan, Turpan, Altay Prefecture, Da Hinggan Ling Prefecture, Ezhou, Chongzuo, Ankang, Shangluo, Zhangjiajie, Haidong, Jiayuguan, Fangchenggang, Nanyang, Zhoushan, Tongchuan, Zhumadian, Luohe, Tibetan Autonomous Prefecture of Garzê, Rikaze, Bortala Mongol Autonomous Prefecture, Naqu, Dai Autonomous Prefecture of Xishuangbanna, Kizilsu Kirghiz Autonomous Prefecture, Diqing Tibetan Autonomous Prefecture, Nujiang of the Lisu Autonomous Prefecture, Tibetan Qiang Autonomous Prefecture of Ngawa, Lhoka, Yushu Tibetan Autonomous Prefecture, Gannan Tibetan Autonomous Prefecture, Tibetan Autonomous Prefecture of Hainan, Qamdo, Haixi Mongolian and Tibetan Autonomous Prefecture, Tibetan Autonomous Prefecture of Huangnan, Tibetan Autonomous Prefecture of Haibei, Nyingchi, Ngari Prefecture, Tibetan Autonomous Prefecture of Golog, Alxa League
